# ﻿To be or not to be… Integrative taxonomy and species delimitation in the daddy long-legs spiders of the genus *Physocyclus* (Araneae, Pholcidae) using DNA barcoding and morphology

**DOI:** 10.3897/zookeys.1135.94628

**Published:** 2022-12-12

**Authors:** Samuel Nolasco, Alejandro Valdez-Mondragón

**Affiliations:** 1 Posgrado en Ciencias Biológicas (Doctorado), Centro Tlaxcala de Biología de la Conducta (CTBC), Universidad Autónoma de Tlaxcala (UATx), Carretera Federal Tlaxcala-Puebla, Km. 1.5, C. P. 90062, Tlaxcala, Mexico; 2 Laboratory of Arachnology (LATLAX), Laboratorio Regional de Biodiversidad y Cultivo de Tejidos Vegetales (LBCTV), Instituto de Biología, Universidad Nacional Autónoma de México (UNAM), sede Tlaxcala, Ex-Fábrica San Manuel, San Miguel Contla, 90640 Santa Cruz Tlaxcala, Tlaxcala, Mexico; 3 CONACYT (Investigador por México), Laboratory of Arachnology (LATLAX), Laboratorio Regional de Biodiversidad y Cultivo de Tejidos Vegetales (LBCTV), Instituto de Biología, Universidad Nacional Autónoma de México (UNAM), sede Tlaxcala, Ex-Fábrica San Manuel, San Miguel Contla, 90640 Santa Cruz Tlaxcala, Tlaxcala, Mexico

**Keywords:** Arteminae, cellar spiders, molecular markers, molecular methods, North America

## Abstract

Integrative taxonomy is crucial for discovery, recognition, and species delimitation, especially in underestimated species complex or cryptic species, by incorporating different sources of evidence to construct rigorous species hypotheses. The spider genus *Physocyclus* Simon, 1893 (Pholcidae, Arteminae) is composed of 37 species, mainly from North America. In this study, traditional morphology was compared with three DNA barcoding markers regarding their utility in species delimitation within the genus: 1) Cytochrome c Oxidase subunit 1 (CO1), 2) Internal Transcribed Spacer 2 (ITS2), and 3) Ribosomal large subunit (28S). The molecular species delimitation analyses were carried out using four methods under the corrected *p*-distances Neighbor-Joining (NJ) criteria: 1) Automatic Barcode Gap Discovery (ABGD), 2) Assemble Species by Automatic Partitioning (ASAP), 3) General Mixed Yule Coalescent model (GMYC), and 4) Bayesian Poisson Tree Processes (bPTP). The analyses incorporated 75 terminals from 22 putative species of *Physocyclus*. The average intraspecific genetic distance (*p*-distance) was found to be < 2%, whereas the average interspecific genetic distance was 20.6%. The ABGD, ASAP, and GMYC methods were the most congruent, delimiting 26 or 27 species, while the bPTP method delimited 33 species. The use of traditional morphology for species delimitation was congruent with most molecular methods, with the male palp, male chelicerae, and female genitalia shown to be robust characters that support species-level identification. The barcoding with CO1 and 28S had better resolution for species delimitation in comparison with ITS2. The concatenated matrix and traditional morphology were found to be more robust and informative for species delimitation within *Physocyclus*.

## ﻿Introduction

Species delimitation is the act of identifying species-level biological diversity (Carstens et al. 2013). It arises from the need to classify, identify, and establish the limits between species (DeSalle et al. 2005; Carstens et al. 2013; Rannala and Yang 2020). The assignment of individual organisms into pre-existing species or higher-level categories (e.g., genus, family, order, etc.) and the designation of new species with proper diagnoses to distinguish them were, for a long-time, roles performed by taxonomist using only traditional morphology and/or somatic characters (Jörger and Schrödl 2013; Rannala and Yang 2020). However, the presence of plastic characters (i.e., characters with high morphological variation), relatively few distinctive morphological traits among species, and morphological stasis due to environmental selection in some groups of organisms (Bickford et al. 2007; Carstens et al. 2013), often makes species delimitation using only morphological evidence extremely difficult or impossible (DeSalle et al. 2005; Rannala and Yang 2020). Spiders are no exception, and due to the complications of using only morphology to identify and delimit species in some groups of araneomorphs and mygalomorphs spiders, different molecular methods have been applied to delimit spider species (Huber et al. 2005; Galtier et al. 2009; Hamilton et al. 2011; Ortiz and Francke 2016; Candia-Ramírez and Francke 2020; Navarro-Rodríguez and Valdez-Mondragón 2020; Valdez-Mondragón 2020; Hazzi and Hormiga 2021). Studies using DNA and RNA have been successful in classifying difficult groups and uncovering underestimated biodiversity (Fox et al. 1977; Wayne et al. 1987; Wilson 1995; Bond 2004).

The spiders of the family Pholcidae C. L. Koch, commonly known as cellar spiders or daddy long-legs spiders, is currently composed of 1,896 species in 97 genera (WSC 2022). Pholcidae is the ninth largest spider family in the World and the most diverse within the Synspermiata clade. The family is composed of five subfamilies: Arteminae Simon, 1893, Modisiminae Simon, 1893, Ninetinae Simon, 1890, Pholcinae C. L. Koch, 1850, and Smeringopinae Simon, 1893 (Huber 2011; Dimitrov et al. 2013; Eberle et al. 2018; Huber and Carvalho 2019). Arteminae includes 107 species distributed in nine genera: *Artema* Walckenaer, 1837, *Aucana* Huber, 2000, *Chisosa* Huber, 2000, *Holocneminus* Berland, 1942, *Nita* Huber & El-Hennawy, 2007, *Pholcitrichocyclus* Ceccolini & Cianferoni, 2022, *Physocyclus* Simon, 1893, *Tibetia* Zhang, Zhu & Song, 2006, and *Wugigarra* Huber, 2001 (WSC 2022).

*Physocyclus* comprises 37 described species, classified into two species groups proposed by Valdez-Mondragón (2013, 2014). To date, the *globosus*-group includes 15 species, while 22 species are recognized in the *dugesi*-group (Valdez-Mondragón 2013, 2014; Jiménez and Palacios-Cardiel 2013; Nolasco and Valdez-Mondragón 2020, 2022). Recently, five species from Mexico were described by Nolasco and Valdez-Mondragón (2020, 2022) based only on traditional morphology, four from the *globosus*-group and one of the *dugesi*-group. The genus is distributed mainly in arid and semiarid ecosystems, as well as tropical dry forests, mostly in North America, with some species in Central America (Valdez-Mondragón 2010, 2013, 2014; Jiménez and Palacios-Cardiel 2013; Nolasco and Valdez-Mondragón 2020, 2022). Most species in the genus are found under 1900 m a.s.l., with the exception of *Physocyclusdugesi* Simon 1893 and *Physocyclusglobosus* (Taczanowski, 1874), whose distributions are influenced by synanthropic activities that have allowed them to occupy higher elevations in association with human dwellings (Valdez-Mondragón 2010). *Physocyclusglobosus* is considered a cosmopolitan species, with records from North and Central America, the Caribbean Islands, the Pacific Islands, South America, Asia, Africa, and Oceania (Valdez-Mondragón 2010; WSC 2022).

The most recent taxonomic revisions and morphological phylogenetic analyses (Valdez-Mondragón 2010, 2013, 2014), as well as new species descriptions using traditional morphology (Jiménez and Palacios-Cardiel, 2013; Nolasco and Valdez-Mondragón 2020, 2022) have revealed 20 new species from Mexico, which represents 54% of the known species diversity in *Physocyclus*.

The general morphology among the different genera of pholcid spiders is conservative, with slight differences in somatic structures. However, primary sexual structures such as male palps and female genitalia, as well as secondary sexual structures such as male chelicerae, are important features used for identification and species diagnosis due to the fact that genitalia evolve more rapidly than non-genital morphological features (Huber 2003; Huber et al. 2018; Valdez-Mondragón 2020). Speciation in arthropods is associated with marked changes in genital morphology, which explains the usefulness of genitalia in distinguishing closely related species (Huber et al. 2005). Sexual structures have different intraspecific and interspecific variation rates in spiders (Eberhard 1985; Eberhard et al. 1998). However, some groups show an overlapping of characters due to minimal morphological variation, making the identification and delimitation of species difficult (Hamilton et al. 2011; Zhang and Li 2014; Ortiz and Francke 2016; Tyagi et al. 2019; Valdez-Mondragón et al. 2019; Candia-Ramírez and Francke 2020; Navarro-Rodríguez and Valdez-Mondragón 2020; Hazzi and Hormiga 2021). Additionally, speciation without changes in genital shape has been recorded in some pholcid spiders, as demonstrated by Huber et al. (2005) with two species of “*Psilochorus*” from South America that showed the same genitalia shape but extreme differences in size and coloration pattern.

Spiders with generally simple genitalia, such as mygalomorphs and some araneomorphs (Synspermiata), are complicated cases for species delimitation and identification using morphology (Huber et al. 2005; Hamilton et al. 2011; Huber and Dimitrov 2014; Valdez-Mondragón 2013, 2014, 2020; Nolasco and Valdez-Mondragón 2022). Additionally, intrasexual polymorphism has been described among females in some pholcids species. Huber & Pérez-González (2001a, b) described two different epigyne morphotypes in females of *Ciboneyaantraia*. In the same way, Valdez-Mondragón (2010) described discontinued interspecific variation in the epigyne shape in females of *Physocyclusenaulus* Crosby, 1926, with three distinct morphotypes.

Due to the poor morphological variation and simple genitalia in some taxonomic groups, approximations based on DNA barcoding using mitochondrial data are often used to establish limits between species, detect species complexes, and/or discover new species in different spider groups (Barrett and Hebert 2005; DeSalle et al. 2005; Bickford et al. 2007; Galtier et al. 2009; Carstens et al. 2013; Navarro-Rodríguez and Valdez-Mondragón 2020; Rannala and Yang 2020; Valdez-Mondragón 2020). Barcoding based on cytochrome c oxidase subunit 1 (CO1) is the common standard in animal Barcoding (including spiders), and its effectivity and resolution has been tested in many studies (Astrin et al. 2006; Planas and Ribera 2015; Ortiz and Francke 2016; Valdez-Mondragón et al. 2019; Navarro-Rodríguez and Valdez-Mondragón 2020; Valdez-Mondragón 2020). While the mitochondrial marker CO1 seems to be suitable for DNA barcoding, it is susceptible to over- and underestimating the diversity in some cases (Astrin et al. 2006; Ortiz and Francke 2016). As such, it is preferable to complement the use of the CO1 marker with other informative mitochondrial (16S) or nuclear (ITS1 or ITS2) markers, as well as morphological evidence, to obtain better resolution (Astrin et al. 2006; Agnarsson 2010; Planas and Ribera 2015; Ortiz and Francke 2016; Valdez-Mondragón et al. 2019; Navarro-Rodríguez and Valdez-Mondragón 2020). The nuclear molecular marker 28S has been used mainly in phylogenetic analyses, providing high resolution for basal clades (Bruvo-Mađarić et al. 2005; Álvarez-Padilla et al. 2009; Arnedo et al. 2009; Dimitrov and Hormiga 2011; Dimitrov et al. 2013). Due to its low substitution rate, 28S is useful for estimating phylogenetic hypotheses of taxa with very old divergence times (Hillis and Dixon 1991). However, this marker has never been tested in species delimitation analyses within the family Pholcidae.

In modern systematics, integrative taxonomy studies that combine different sources of evidence, such as molecular markers and morphological data, are commonly implemented to help delimit and diagnose new species or even identify cryptic species complexes (Astrin and Stueben 2008; Álvarez-Padilla et al. 2009; Correa-Ramírez et al. 2010; Hamilton et al. 2011; Montes de Oca et al. 2015; Planas and Ribera 2015; Valdez-Mondragón and Francke 2015; Cao et al. 2016; Valdez-Mondragón et al. 2019; Newton et al. 2020; Valdez-Mondragón and Cortez-Roldán 2021). Recent publications on spider taxonomy have additionally used other sources of information, such as Ecological Niche Modeling (ENM), lineal morphology, and even geometric morphology to characterize species (Valdez-Mondragón et al. 2019; Navarro-Rodríguez and Valdez-Mondragón 2020; Solís-Catalán 2020).

From the first proposal for a DNA barcoding initiative using a single locus (the CO1 mitochondrial gene for animals) as a diagnostic for assigning species (Hebert et al. 2003), the field of species delimitation has been refined and improved, incorporating different theories (e.g., coalescence) and methods (DeSalle et al. 2005; Hickerson et al. 2006; Pons et al. 2006; Puillandre et al. 2012, 2021; Carstens et al. 2013; Rannala and Yang 2020). Species delimitation analyses using DNA Barcoding, initially with only a single locus (CO1), is limited as a diagnostic for assigning species based on the fact that intraspecific and interspecific sequence distances may be similar in large populations (Hebert et al. 2003; Rannala and Yang 2020). Therefore, many studies have opted for a multi-locus approach, combining CO1 with other molecular markers to give additional robust evidence for species delimitation (Astrin et al. 2006; Agnarsson 2010; Planas and Ribera 2015; Ortiz and Francke 2016; Ballesteros and Hormiga 2018; Navarro-Rodríguez and Valdez-Mondragón 2020; Hazzi and Hormiga 2021).

The aim of this study is to carry out different species delimitation methods within the spider genus *Physocyclus* under an integrative taxonomic approach. To carry out this, we use a combination of molecular markers (CO1, ITS2, and 28S) and traditional morphology of diagnostical features (e.g., male palps, male chelicerae, and female epigynes) to test the validation of the currently recognized species within the genus.

## ﻿Materials and methods

### ﻿Biological material

Specimens were provided by the Laboratory of Arachnology (**LATLAX**) **IB-UNAM**, Tlaxcala, Mexico; the Colección Nacional de Arácnidos (**CNAN**), Institute of Biology, Universidad Nacional Autónoma de México (**IB-UNAM**), Mexico City; Centro de Investigaciones Biológicas de Noreste (**CIBNOR**), La Paz, Baja California Sur, Mexico; and Colección Aracnológica de la Facultad de Biología de la Universidad Michoacana de San Nicolás de Hidalgo (**CAFBUM**), Michoacan, Mexico. Specimens were preserved in 80% ethanol for morphological studies and 96% ethanol for molecular studies. Female sexual structures (epigyne) were dissected in 80% ethanol and cleaned with potassium hydroxide (10% KOH). This to remove all soft tissue and observe with clarity the taxonomically important internal structures, such as the pore plates. The left male palps were dissected and observed in 80% ethanol. Structures were photographed submerged in commercial gel alcohol to hold them in the appropriate position, while the preparation was done with structures completely covered with 80% ethanol. Specimen observations and identifications were carried out using a Zeiss Discovery V8 stereo microscope. A Zeiss Axiocam 506 color camera attached to a Zeiss AXIO Zoom V16 stereo microscope was used to photograph focal structures (male palps, female epigynes, and male chelicerae). Digital images of morphological structures were edited in Adobe Photoshop CS6.

### ﻿Taxon sampling

The molecular analyses were based on a total of 194 sequences of 23 putative species. Species used in the molecular analyses are listed in Table 1. The ingroup includes 188 sequences of 22 species of *Physocyclus* previously described by Valdez-Mondragón (2010, 2013, 2014) and Nolasco and Valdez-Mondragón (2020, 2022). The CO1 sequence matrix is composed of 75 sequences (22 species), ITS2 with 55 sequences (20 species), and 28S with 58 sequences (21 species). Four different partitions were used in the analyses: 1) CO1: 642 bp, 2) ITS2: 505 bp, 3) 28S: 891 bp, and 4) the concatenated matrix CO1+ITS2+28S: 2038 bp. Since this study is focused solely on species delimitation within *Physocyclus* and not on the molecular dating and phylogenetic relationships within Arteminae, only *Chisosa* sp. (Arteminae) was used as an outgroup, to root the trees in the various analyses.

**Table 1.** Specimens sequenced for each species of *Physocyclus*, DNA voucher numbers, localities, and GenBank accession numbers for CO1, ITS2, and 28S. Mexican state abbreviations: BC, Baja California; BCS, Baja California Sur; COL, Colima; GRO, Guerrero; HGO, Hidalgo; JAL, Jalisco; MICH, Michoacán, OAX, Oaxaca; PUE, Puebla. **Non-Mexican localities.

Species	DNA Code LATLAX	Locality (Mexico)	CO1	ITS2	28S
*P.bicornis*	Ara0394	GRO: Copala	OP293157	OP296540	OP295410
*P.bicornis*	Ara0396	GRO: Quechultenango	OP293158	OP296538	OP295411
*P.bicornis*	Ara0398	GRO: Coyuca	OP293159	OP296539	
*P.bicornis*	Ara0445	GRO: Quechultenango	OP293160	OP296541	OP295412
*P.brevicornus*	Ara0515	JAL: Cocula	OP293161	OP296542	OP295413
*P.brevicornus*	Ara0516	MICH: Morelia	OP293162		
*P.brevicornus*	Ara0518	MICH: Morelia	OP293163	OP296543	OP295414
*P.cornutus*	Ara0405	BCS: Los Cabos	OP293164		OP295415
*P.cornutus*	Ara0406	BCS: Los Cabos	OP293165		OP295416
*P.dugesi*	Ara0597	HGO: Tula	OP293166	OP296544	OP295417
*P.dugesi*		Costa Rica**	AY560787		AY560750
*P.enaulus*	Ara0391	COA: Saltillo	OP293167	OP296545	OP295418
*P.enaulus*	Ara0392	COA: Saltillo	OP293168	OP296546	OP295419
*P.enaulus*	Ara0393	COA: Saltillo	OP293169	OP296547	OP295420
*P.enaulus*		U.S.A.**	MG268722		
*P.franckei*	Ara0378	HGO: Tolantongo	OP293170	OP296548	
*P.franckei*	Ara0379	HGO: Cárdenas	OP293171	OP296549	OP295421
*P.franckei*	Ara0381	HGO: Cardonal	OP293172	OP296550	
*P.franckei*	Ara0382	HGO: Cardonal	OP293173	OP296551	
*P.gertschi*	Ara0575	GRO: José Azueta	OP293174	OP296552	OP295422
*P.gertschi*	Ara0576	GRO: José Azueta	OP293175	OP296553	OP295423
*P.gertschi*	Ara0577	GRO: José Azueta	OP293176	OP296554	OP295424
*P.globosus*	Ara0473	COL: Coquimatlán	OP293177	OP296555	OP295425
*P.globosus*	Ara0533	BCS: Comundú	OP293178		
*P.globosus*	Ara0535	GRO: Técpan	OP293179	OP296556	OP295426
*P.globosus*		Quintana Roo	MT888253		
*P.globosus*		Cuba**	AY560788		AY560751
*P.lautus*	Ara0459	MICH: Cárdenas	OP293180	OP296557	OP295427
*P.lautus*	Ara0579	MICH: Coahuayana	OP293181	OP296558	OP295428
*P.lautus*	Ara0583	JAL: La Huerta	OP293182		OP295429
*P.lyncis*	Ara0437	JAL: Zapopan	OP293183	OP296559	OP295430
*P.lyncis*	Ara0754	JAL: Zapopan	OP293184	OP296560	OP295431
*P.mariachi*	Ara0745	JAL: Hostotipaquillo	OP293185	OP296561	OP295432
*P.mariachi*	Ara0746	JAL: Hostotipaquillo	OP293186	OP296562	OP295433
*P.mariachi*	Ara0748	JAL: Plan de Barrancas	OP293187	OP296564	OP295434
*P.merus*	Ara0898	SLP: Villa de Reyes	OP293188	OP296565	OP295435
*P.merus*	Ara0915	SLP: Villa de Reyes	OP293189		OP295436
*P.merus*	Ara0916	SLP: Villa de Reyes	OP293190	OP296566	OP295437
*P.merus*	Ara0917	SLP: Villa de Reyes	OP293191	OP296567	OP295438
*P.merus*	Ara0918	SLP: Villa de Reyes	OP293192		OP295438
*P.michoacanus*	Ara0585	MICH: Tzitzio	OP293193	OP296568	OP295440
*P.michoacanus*	Ara0586	MICH: Tzitzio	OP293194	OP296569	OP295441
*P.michoacanus*	Ara0598	JAL: Jilotlán	OP293195		OP295442
*P.modestus*	Ara0467	PUE: Miahuatlán	OP293196	OP296570	OP295443
*P.modestus*	Ara0469	GRO: Tepecoacuilco	OP293197	OP296571	OP295444
*P.modestus*	Ara0480	GRO: Escudero	OP293198		OP295445
*P.modestus*	Ara0482	GRO: Quechultenango	OP293199		
*P.mysticus*	Ara0450	BC: Ensenada	OP293200	OP296572	
*P.mysticus*	Ara0451	BC: Ensenada	OP293201	OP296573	
*P.mysticus*	Ara0452	BCS: Mulegé	OP293202		OP295446
*P.mysticus*	Ara0453	BCS: Mulegé	OP293203		OP295447
*P.mysticus*	Ara0524	BC: Ensenada	OP293204		
*P.paredesi*	Ara0483	OAX: Tadela	OP293205	OP296574	OP295448
*P.paredesi*	Ara0484	OAX: Tadela	OP293206	OP296575	OP295449
*P.paredesi*	Ara0485	OAX: Totolapa	OP293207	OP296576	OP295450
*P.paredesi*	Ara0486	OAX: Totolapa	OP293208		OP295451
*P.pocamadre*	Ara0371	BCS: Mulegé	OP293209		
*P.reddelli*	Ara0487	HGO: Araya	OP293210	OP296577	OP295452
*P.reddelli*	Ara0488	HGO: Araya	OP293211	OP296578	
*P.rothi*	Ara0383	BCS: Comundú	OP293212	OP296579	OP295453
*P.rothi*	Ara0384	BCS: Comundú	OP293213	OP296580	OP295456
*P.rothi*	Ara0386	BCS: La Paz	OP293214		OP295454
*P.rothi*	Ara0387	BCS: La Paz	OP293215		OP295455
*P.sikuapu*	Ara0749	MICH: Costa Aquila	OP293216	OP296581	OP295457
*P.sikuapu*	Ara0750	MICH: Costa Aquila	OP293217	OP296582	OP295458
*P.sikuapu*	Ara0751	MICH: Costa Aquila	OP293218	OP296583	OP295459
*P.sikuapu*	Ara0752	MICH: Costa Aquila	OP293219	OP296584	OP295460
*P.validus*	Ara0502	COL: Coquimatlán	OP293220	OP296585	
*P.validus*	Ara0503	GRO: Eduardo Neri	OP293221	OP296586	OP295461
*P.xerophilus*	Ara0372	BCS: Mulegé	OP293222	OP296587	OP295464
*P.xerophilus*	Ara0373	BCS: Mulegé	OP293223	OP296588	OP295462
*P.xerophilus*	Ara0374	BCS: Mulegé	OP293224	OP296589	OP295465
*P.xerophilus*	Ara0375	BCS: Mulegé	OP293225	OP296590	
*P.xerophilus*	Ara0376	BCS: Mulegé	OP293226	OP296591	OP295463
*P.xerophilus*	Ara0377	BCS: Mulegé	OP293227	OP296592	
*Chisosa* sp.	Ara0454	PUE: Miahuatlán	OP293228	OP296593	OP295466
*Chisosa* sp.	Ara0455	PUE: Miahuatlán	OP293229	OP296594	OP295467

### ﻿DNA extraction, amplification, and sequencing

For DNA extraction, we used the Qiagen DNeasy extraction kit, following the modifications suggested by Valdez-Mondragón and Francke (2015) and Valdez-Mondragón (2020). Three legs from each specimen were used for DNA extraction, using males, females, and juveniles, depending on the available specimens per species. The criterion for selecting tissues was based on tissue antiquity, considering only specimens collected in the last five years in order to extract high DNA quality. Amplification of the CO1 locus was carried out using two different primer sets: LCO1490/HCO2198 and LCO-JJ/HCO-JJ; for ITS2, the primer set 5.8S and CAS28SB1d was used; and for 28S, 28S-B1 and 28S-B2 (Table 2). Polymerase chain reactions (PCR) were carried out in a Verity-Applied Biosystems 96 Well Thermal Cycler. The final volume of each PCR tube was 20 µl: 2.3 µl injectable H_2_O, 2.0 µl Q-solution, 10 µl Multiplex-Mix PCR, 1.6 µl of each primer (forward and reverse), and 2.5 µl of extracted DNA sample. The cycles and optimal temperatures for CO1 and ITS2 amplification were as follows: Initial heating phase of 15 min at 95 °C, 35 amplification cycles of 35 s at 94 °C (denaturing), 1 min 30 s at 40 °C (alignment), and 1 min 30 s at 72 °C (elongation), with a final elongation of 10 min at 72 °C. Two different protocols were used to amplify the 28S region. The first followed Eberle et al. (2018): initial heating phase of 15 min at 95 °C, 35 amplification cycles of 35 s at 95 °C (denaturing), 1 min at 51 °C (alignment), and 1 min at 72 °C (elongation), with a final elongation of 10 min at 72 °C. The second protocol is a modification of Eberle et al. (2018): initial heating phase of 15 min at 95 °C, 35 amplification cycles of 35s at 94 °C (denaturing), 1 min 30 s at 59 °C (alignment), and 1 min 30 s at 72 °C (elongation), with a final elongation of 10 min at 72 °C. Gel electrophoresis was carried out with 0.5% agarose using the molecular weight marker Perfect DNA 100 bp Ladder Novage to calculate fragment size of amplifications. Gels were visualized in a photodoc BioDoc-It2 Imager 315 Imaging System LMS-20 Transilluminator. PCR products were purified using a QIAquick Qiagen purification kit. Tissue selection, DNA extraction, amplification, and purification were performed at the Laboratory of Molecular Biology at Laboratorio Regional de Biodiversidad y Cultivo de Tejidos Vegetales (**LBCTV**), IB-UNAM, Tlaxcala City. Sanger sequencing was done at the Laboratory of Molecular Biology and Health, IB-UNAM, Mexico City.

**Table 2.** Primer sets used in this study for PCR amplification.

Molecular marker	Primer	Sequence (5’-3’)	Author
CO1	HCO2198	TAAACTTCAGGGTGACCAAAAAATC	Folmer et al. (1994)
	LCO1490	GGTCAACAAATCATAAAGATATTGG	
	HCO-JJ	AWACTTCVGGRTGCVCAAARAATCA	Astrin and Stueben (2008)
	LCO-JJ	CHACWAAYCATAAAGATATYGG	
ITS2	5.8S	CGCCTGTTTATCAAAAACAT	Ji et al. (2003); Planas and Ribera (2014)
	CAS28sB1d	TTC TTT TCC TCC SCT TAY TRA TAT GCT TAA	
28S	28S-B1	GACCGATAGCAAACAAGTACCG	Bruvo-Mađarić et al. (2005)
	28S-B2	CACGGGTCGATGAAGAACGC	

### ﻿DNA sequence alignment and editing

Both forward and reverse DNA strands were sequenced. DNA sequences were edited in Geneious v. 8.1.9 (Rozen and Skaletsky 2000). Multiple alignment of sequences was implemented using MAFFT v. 7 (Katoh and Toh 2008) through online platform (https://mafft.cbrc.jp/alignment/server/), with the following commands: Auto (FFT-NS-2, FFTNS-I or L-INS-I, depending on data size). In some cases, alignment was done manually and edited with BioEdit v. 7.0.5.3 (Hall 1999). The concatenated matrix (COI+ITS2+28S) was built in Geneious v. 8.1.9. The aligned matrices were subsequently used in the molecular analyses.

### ﻿Molecular analysis and species delimitation

Four different molecular delimitation methods were used under the corrected *p*-distances Neighbor-Joining (NJ) criteria: 1) ABGD (Automatic Barcode Gap discovery) (Puillandre et al. 2012), 2) ASAP (Assemble Species by Automatic Partitioning) (Puillandre et al. 2021), 3) GMYC (General Mixed Yule Coalescent) (Pons et al. 2006), and 4) bPTP (Bayesian Poisson Tree Process) (Zhang et al. 2013; Kapli et al. 2017).

### ﻿p-distances under Neighbor-Joining (NJ)

The genetic distance tree was reconstructed with MEGA v. 10.1.7 (Kumar et al. 2016) under the following parameters: Number of replicates = 1000, Bootstrap support values = 1000 (significant values ≥ 50%), Substitution type = nucleotide, Model = *p*-distance, Substitution to include = d: Transitions + Transversions, Rates among sites = Gamma distributed with invariant sites (G+I), Missing data treatment = Pairwise deletion.

### ﻿Automatic Barcode Gap Discovery (ABGD)

This method aims to find gaps in genetic divergence, considering that intraspecific genetic variation is theoretically smaller than interspecific divergences. It first generates a prior partition of the data into putative species (initial partitions, IP). Then, these initial partitions are recursively partitioned until there is no further partitioning of the data (recursive partitions, RP). ABGD analyses were carried out on the online platform (https://bioinfo.mnhn.fr/abi/public/abgd/) using the following options: K2P distances non-corrected, P_min_ = 0.001, P_max_ = 0.1, Steps = 10, Relative gap width (X) = 1, Nb bins = 20.

### ﻿Assemble Species by Automatic Partitioning (ASAP)

This is an ascending hierarchical clustering method. Sequences are merged into groups that are successively merged further until all sequences form a single group. Partitions are equivalent to each sequence merge step. The software analyzes all partitions and scores the most probable groups into a tree (Puillandre et al. 2021). ASAP analyzes were run on the online platform (https://bioinfo.mnhn.fr/abi/public/asap/) using Kimura (K80) distance matrices and configured under following parameters: Substitution model = *p*-distances, Probability = 0.01, Best scores = 10, Fixed seed value = -.

### ﻿General Mixed Yule Coalescent (GMYC)

This approach applies single (Pons et al. 2006) or multiple (Monaghan et al. 2009) time thresholds to delimit species in a Maximum Likelihood context, using ultrametric trees as input (Ortiz and Francke 2016). Ultrametric trees were generated with phylogenetic analyses in the BEAUti and BEAST v. 1.10.4 software (Drummond et al. 2002–2018) using a coalescent (constant population) tree prior. An independent log normal uncorrelated clock was applied to each partition with their respective evolution model and substitution rates (CO1: GTR + I + G; ITS2: K2P; 28S: GTR + I + G). Five independent analyses were run, each with 40 million iterations. Tracer 1.6 (Rambaut and Drummond 2003–2013) was used to evaluate convergence values, with the ESS (Effective Sample Size) > 200. Tree Annotator 2.6.0 (a BEAST package) was used to construct maximum credibility of clades trees, after discarding the first 25% of each independent run as burn-in. Finally, the GMYC method was implemented in the web platform (https://species.h-its.org/gmyc/), which uses the original R implementation of the GMYC model (Fujisawa and Barraclough 2013).

### ﻿Bayesian Poisson Tree Processes (bPTP)

This method is similar to GMYC, but does not require an ultrametric tree as input because the models of speciation rate are implemented directly using the number of substitutions calculated from branch lengths. The Bayesian and Maximum Likelihood variants were carried out on the online platform (https://species.h-its.org/ptp/), with the following options: Rooted tree, MCMC = 1000000, Thinning = 100, Burn-in = 0.1, Seed = 123. The resulting trees were edited with the iTOL online version (https://itol.embl.de/) (Letunic and Bork 2021) and Photoshop CS6. To delimit different species, we used the congruence integration criteria. It is based on the correspondence among the different molecular methods to generate a highly supported species hypothesis (DeSalle et al. 2005; Hamilton et al. 2011; Navarro-Rodríguez and Valdez-Mondragón 2020; Valdez-Mondragón 2020).

## ﻿Results

### ﻿Molecular analyses of genetic distances

**Figure 1.**
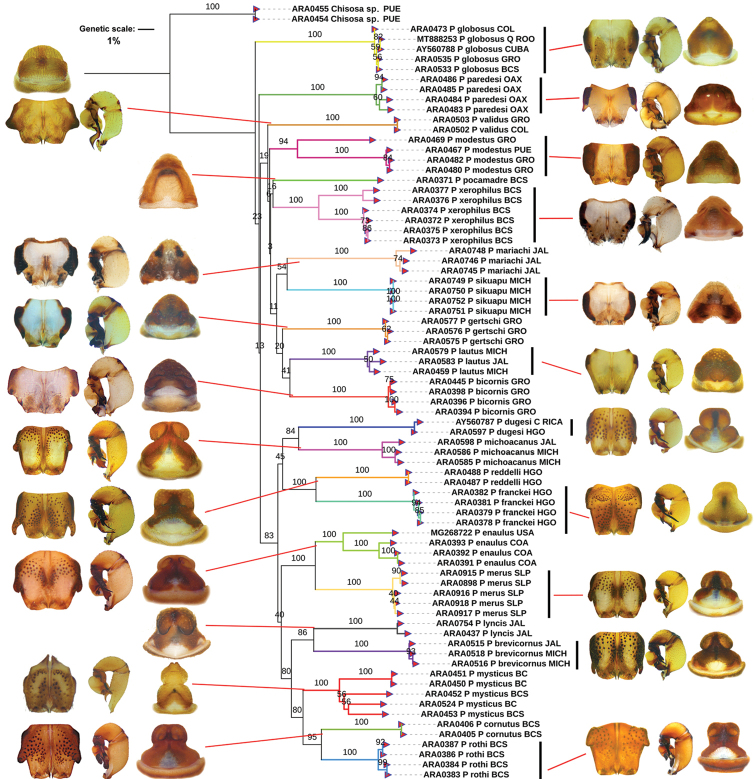
Neighbor-Joining (NJ) with corrected *p*-distances tree constructed with CO1 barcode sequences from different species of *Physocyclus*. Branch colors indicate putative species. Male chelicerae, male palps, and female epigynes are shown for each species. Numbers above branches represent significant Bootstrap support values (> 50%).

**Figure 2.**
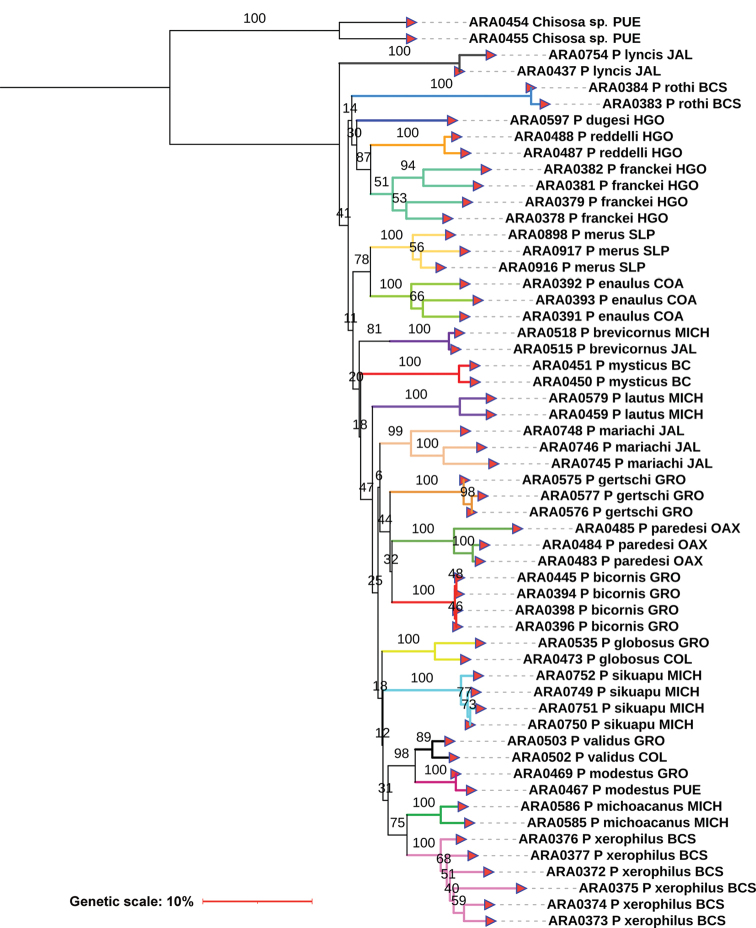
Neighbor-Joining (NJ) with corrected *p*-distances tree constructed with ITS2 barcode sequences from different specimens and species of *Physocyclus*. Branch colors indicate putative species. Numbers above branches represent significant Bootstrap support values (> 50%).

**Figure 3.**
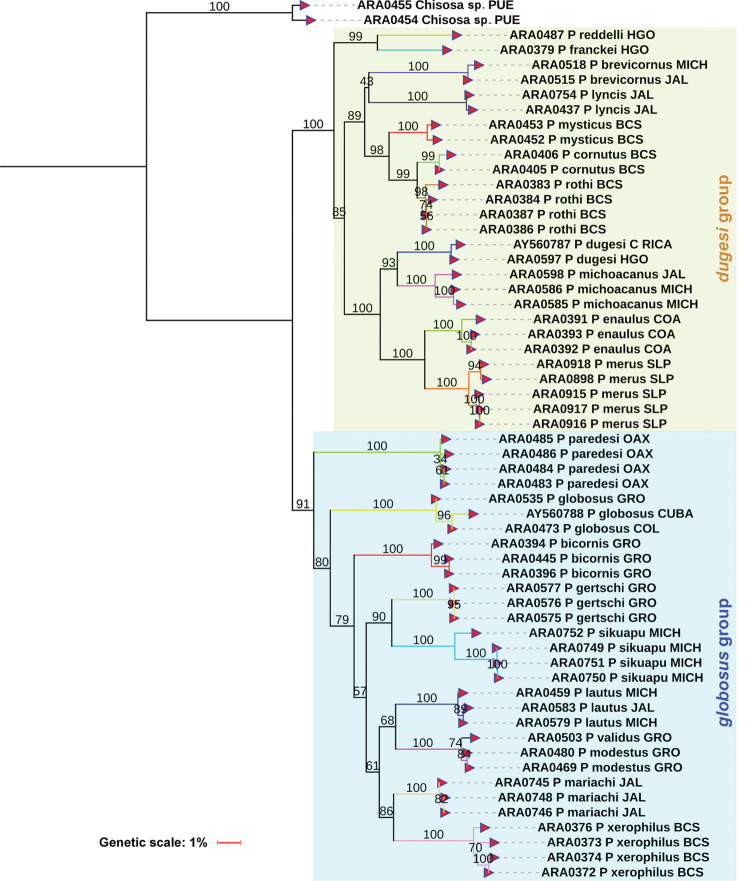
Neighbor-Joining (NJ) with corrected *p*-distances tree constructed with 28S barcode sequences from different specimens and species of *Physocyclus*. Branch colors indicate putative species. Numbers above branches represent significant Bootstrap support values (> 50%).

The corrected *p*-distances under NJ using the CO1 matrix recovered 22 species of *Physocyclus*. This is concordant with the morphology analysis of features commonly used to identify and diagnose at the species level (Valdez-Mondragón 2013, 2020; Valdez-Mondragón and Francke 2015) (Fig. 1). Both the morphology and all genetic distance analyses using CO1 recovered groups that correspond with a described species. The NJ analyses using the ITS2 marker recovered 20 species (Fig. 2), whereas analyses with 28S recovered 21 species (Fig. 3). The average genetic *p*-distances among *Physocyclus* species were 16.4% (min: 14.89%, max: 17.96%) for CO1, 29.4% (min: 25.37%, max: 38.59%) for ITS2, and 14.4% (min: 14.15%, max: 17.87%) for 28S (Figs 1–3, Table 3). The average intraspecific distances using CO1 were below 2% for most species (18/22). However, four species (*P.enaulus* Crosby, 1926, *P.modestus* Gertsch, 1971, *P.mysticus* Chamberlin, 1924, and *P.xerophilus* Nolasco & Valdez-Mondragón, 2020) showed average intraspecific genetic distances between 4–6% (Table 3), with high Bootstrap support in each case (> 94%) (Fig. 1). The Bootstrap support values for all species were high (100%) (Fig. 1). The *globosus* and *dugesi* species groups were not recovered in the CO1 topology. In the ITS2 tree, six species (*P.bicornis* Gertsch, 1971, *P.lautus* Gertsch, 1971, *P.lyncis* Nolasco & Valdez-Mondragón, 2022, *P.modestus*, *P.mysticus*, and *P.validus* Gertsch, 1971) had average intraspecific genetic distances below 2%, while the rest showed average intraspecific genetic distances over 2% (Fig. 2). Bootstrap support values for species in the ITS2 topology were significant (over 89%), except for *P.franckei* (51%). Using the 28S marker, average intraspecific genetic distances for all species were below 2% (Fig. 3) with high Bootstrap support values (> 95%). The 28S tree was the only one to recover both the *globosus* and *dugesi* species groups with high Bootstrap support values (100% for the *dugesi* group and 91% for the *globosus* group) (Fig. 3).

**Table 3.** Average CO1 genetic distances (*p*-distances) among *Physocyclus* species.

Species	1	2	3	4	5	6	7	8	9	10	11	12	13	14	15	16	17	18	19	20	21	22
**1. *P.bicornis***	0.7																					
**2. *P.brevicornus***	18.4	0.3																				
**3. *P.cornutus***	16.4	14.1	0.2																			
**4. *P.dugesi***	20.3	17.1	18.5	0.5																		
**5. *P.enaulus***	17.6	17.3	15.3	17.6	4.5																	
**6. *P.franckei***	20.3	17.7	17.9	18.2	17.4	0.3																
**7. *P.gertschi***	13.8	18.3	17.1	20.1	19.7	19.4	0.2															
**8. *P.globosus***	18.3	19.5	18.4	18.4	16.5	18.9	16.8	0.4														
**9. *P.lautus***	12.9	17.4	15.9	17.8	16.9	18.4	14.3	17.1	1.4													
**10. *P.lyncis***	18.0	12.7	15.8	17.3	16.2	19.1	18.3	18.3	17.3	1.1												
**11. *P.mariachi***	16.4	20.1	20.1	18.4	20.0	19.2	16.5	19.6	15.6	18.3	1.5											
**12. *P.merus***	17.6	17.2	15.8	17.9	11.0	19.7	19.1	17.9	16.9	15.5	19.4	0.9										
**13. *P.michoacanus***	18.3	17.0	17.2	14.7	15.0	16.6	17.4	16.7	15.3	16.3	20.3	17.0	1.9									
**14. *P.modestus***	15.6	19.1	17.6	18.8	16.4	18.5	16.1	15.8	14.1	18.9	17.9	17.9	17.1	5.8								
**15. *P.mysticus***	18.2	14.3	12.8	16.0	15.0	16.9	17.4	16.9	16.2	15.3	17.8	14.6	15.8	16.9	5.7							
**16. *P.paredesi***	18.0	19.5	19.2	18.5	17.0	19.4	17.0	17.5	16.3	18.0	18.9	18.2	19.8	17.8	17.6	1.3						
**17. *P.pocamadre***	15.8	18.7	16.5	19.6	16.6	19.8	16.3	16.9	13.8	16.1	15.8	16.4	17.9	15.1	15.4	16.4	0.0					
**18. *P.reddelli***	19.2	17.5	18.5	17.1	16.6	13.0	19.2	19.4	17.5	16.8	18.6	17.0	17.1	18.4	15.8	17.7	16.9	0.0				
**19. *P.rothi***	16.0	13.0	9.7	16.4	14.3	14.6	14.2	17.0	15.6	14.6	17.3	15.5	14.9	16.6	11.0	17.8	16.5	17.5	0.8			
**20. *P.sikuapu***	16.6	17.9	18.5	18.3	18.2	19.1	14.5	18.3	15.7	17.1	15.1	18.1	19.0	16.0	16.8	16.8	15.6	16.6	16.6	0.0		
**21. *P.validus***	16.7	20.0	19.5	20.4	18.9	19.5	16.1	17.4	16.9	18.8	19.0	18.3	17.8	16.8	18.9	17.6	16.2	18.9	19.5	16.8	0.0	
**22. *P.xerophilus***	15.9	18.6	17.7	19.3	17.1	17.9	14.3	15.8	13.9	18.2	15.7	16.4	18.1	14.8	16.9	15.8	13.7	16.7	17.4	15.3	15.2	4.0

### ﻿Molecular methods for species delimitation

**Figure 4.**
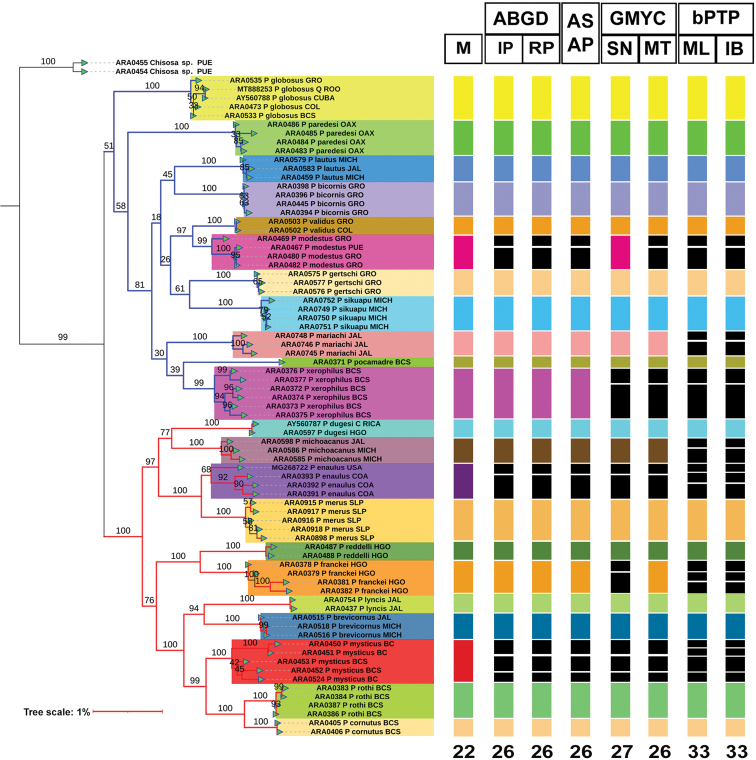
Maximum Likelihood (ML) tree of *Physocyclus* (log likelihood: -3749.87) constructed with the concatenated matrix (CO1+ITS2+28S). Bar colors represent putative species in the tree and in the columns, which represent the different species delimitation methods analyzed. Branch colors represent species groups: *globosus* (blue) and *dugesi* (red). Numbers below the columns represent the species recovered in each species delimitation method (not considering *Chisosa* sp.). Numbers above branches represent Bootstrap support values for ML (> 50% significant). Column abbreviations: Morphology (M); ABGD with initial (IP) and recursive (RP) partitions; ASAP; GMYC with single (SN) and multi (MT) thresholds; bPTP with Maximum Likelihood (ML) and Bayesian Inference (IB) variants.

The Maximum Likelihood (ML) tree of the concatenated matrix (CO1+ITS2+28S) (Fig. 4) found congruence among the four different molecular species delimitation methods and using traditional morphology. This was found for 15 species: *Physocyclusbicornis*, *P.brevicornus*, *P.cornutus*, *P.dugesi*, *P.gertschi*, *P.globosus*, *P.lautus*, *P.lyncis*, *P.merus*, *P.paredesi*, *P.pocamadre*, *P.reddelli*, *P.rothi*, *P.sikuapu*, and *P.validus* (Fig. 4). *Physocyclusmariachi* and *P.michoacanus* are recovered as distinct species by most methods except for bPTP, whereas *P.franckei* is not recovered under the bPTP and GMYC methods in the single threshold (SN) (Fig. 4). *Physocyclusenaulus*, *P.modestus*, *P.mysticus*, and *P.xerophilus* were found to contain more than two species (2–4) by most of the species delimitation methods (Fig. 4).

The most congruent methods with morphology were the barcoding method ABGD, ASAP, and GMYC, which delimited 26 (ABGD IP and RP, ASAP, and GMYC MT) and 27 (GMYC SN) putative species, respectively. The most incongruent result of the analyses was bPTP, which delimited 33 putative species under ML and IB variants (Fig. 4).

Both species groups (*globosus* and *dugesi*) were recovered in the ML analysis using the concatenated matrix (CO1+ITS2+28S) (Fig. 4). The *dugesi* group was recovered with significant Bootstrap support (100%), whereas the *globosus* group had Bootstrap support value of 51%.

## ﻿Discussion

Two different approaches (DNA taxonomy and DNA barcoding) were proposed by DeSalle et al. (2005) to overcome several weaknesses of traditional morphology-based taxonomic systematics, and to resolve the crucial need for accurate and rapid species identification tools (Hebert et al. 2003; Tautz et al. 2003). DNA barcoding is useful for recognizing cryptic species (two or more distinct species that are erroneously classified as the same species due to similar morphology) (Bickford et al. 2007). Even only with CO1, DNA barcoding is helpful in species diagnosis due to the fact that sequence divergences are ordinarily much lower among individuals of the same species than between closely related species (Hebert et al. 2004). For some groups of arachnids, traditional morphology fails to recognize and delineate species boundaries. Also, identify sister or cryptic species requires other types or evidence such as molecular data, ecological niche modeling, morphometric morphology, haplotype networks, and biogeographical approximations (Hebert et al. 2003, 2004; Hamilton et al. 2011, 2014; Montes de Oca et al. 2015; Ortiz and Francke 2016; Cruz-López et al. 2019; Valdez-Mondragón et al. 2019; Newton et al. 2020; Valdez-Mondragón and Cortez-Roldán 2021). However, as demonstrated herein, spiders of the genus *Physocyclus* have robust morphology for diagnosis and identification at the species level, mainly of primary and secondary sexual characters, such as chelicerae and palps (males) or epigynes (females).

In the genetic distance analyses performed with independent matrices of CO1, ITS2, and 28S, all the species terminals were recovered. The genetic intraspecific distances for ITS2 were found to be relatively high in the majority of species (over 2%). However, the average intraspecific genetic distances using the CO1 and 28S markers were lower in the majority of species (< 2%). Agnarsson (2010), Valdez-Mondragón et al. (2019), and Navarro-Rodríguez and Valdez-Mondragón (2020) mention that ITS2 is inadequate for resolving relationships between closely related species of spiders. Our data corroborate this, showing the unreliability of this gene for genetic distance (and species delimitation) analyzes when used on its own. However, the use of a concatenated matrix of nuclear (28S and ITS2) and mitochondrial (CO1) markers provides better results with more robust evidence for delimiting species based on molecular data (Astrin et al. 2006; Agnarsson 2010; Planas and Ribera 2015; Ortiz and Francke 2016; Navarro-Rodríguez and Valdez-Mondragón 2020).

When looking at CO1, Pholcid spiders generally show high genetic divergences among species (Astrin et al. 2006). In contrast to reports for spiders in general that show values of 14.4% (Hebert et al. 2003) and 16.4% (Barrett and Hebert 2005), Pholcids’ average interspecific genetic distance for CO1 is 19.8% (Astrin et al. 2006). Our results found a value of average interspecific distance at the CO1 marker observed in *Physocyclus* of 16.4% (Fig. 1, Table 3). This value fits within the average limits of other spiders; however, this tendency in the Pholcidae family is not always the case. The average interspecific genetic distance for CO1 in other genera such as *Ixchela* Huber, 2000 (Modisiminae) were found to be lower, 12% (Valdez-Mondragón 2020). Although most species were included in the analyses (22 of 37), missing species might have an effect and overestimate the average interspecific distances, because perhaps the sister species of each species is not found in the data set. However, the results and topologies were consistent along the analyzes.

With regards to molecular delimitation methods, ABGD used to be sensitive to sampling effect and tended to moderately over-split, as demonstrated in the mygalomorph genera *Aphonopelma* Pocock, 1901 by Hamilton et al. (2014) and *Bonnetina* Vol, 2000 by Ortiz and Francke (2016) and Candia-Ramírez and Francke (2020). Similar results were observed in *Loxosceles* Heineken & Lowe, 1832 (Valdez-Mondragón et al. 2019; Navarro-Rodríguez and Valdez-Mondragón 2020), where the ABGD method generated an inflated number of delimited species. However, within the pholcid genus *Ixchela*, it was observed by Valdez-Mondragón (2020) that the most incongruent method was bPTP (both ML and IB variants), as was also found in the molecular analyses herein with the genus *Physocyclus*.

The most congruent methods that delimited a similar number of species in this study were ABGD, ASAP, and GMYC, which was corroborated by traditional morphology (Fig. 4). The bPTP method delimited a higher number of species in comparison with morphology. These results contrast with those found by Ortiz and Francke (2016), Valdez-Mondragón et al. (2019), and Navarro-Rodríguez and Valdez-Mondragón (2020), in which the bPTP and GMYC analyses were the most congruent methods with the morphology in spiders. This may be due to the inclusion of the 28S marker, which might be causing tree-based analyzes (bPTP) to generate an overestimation in the number of putative species recovered. According to Luo et al. (2018), GMYC and bPTP are negatively influenced by gene flow and are sensitive to the ratio of population size to divergence time, reflecting the important impact of incomplete lineage sorting on species delimitation.

In such cases of incongruency between the molecular methods and morphology, as in *Physocyclusenaulus*, *P.modestus*, *P.mysticus*, and *P.xerophilus*, no significant morphological differences were found within individuals of each species. However, *P.enaulus* was the exception, where Valdez-Mondragón (2010) recorded three different morphotypes of epigyne shape. Unfortunately, we could not to get sequences of all the morphotypes. As Valdez-Mondragón (2010, 2014) mentions, the morphology of somatic and sexual characters in the genus *Physocyclus* is usually highly conserved. Similarly, morphological changes might not be correlated with species boundaries, or may not be useful for delimiting species if interspecific recognition occurs in non-visual signs (chemical or mating calls), or even biogeographical traits (microhabitats). Furthermore, it is possible that the species may be under stabilizing selection that promotes morphological stasis (Bickford et al. 2007).

According with Hamilton et al. (2011), species delimitation based only on molecular data can rarely be achieved, and additional types of evidence such as biogeographical information is needed. In the case of the multiple species inferred within *P.mysticus*, no significative morphological or habitat differences are apparent. All specimens are from xerophytic scrub and present similar microhabitats of living under big boulders on the ground. Sympatric speciation may be possibly, as other species of the genus *Physocyclus* have been collected in the same locality (Valdez-Mondragón 2010, 2013). *Physocyclusmodestus* presents a similar case, being one widespread species from Guerrero, Morelos, Oaxaca, and Puebla. However, habitat differences exist among the different populations of this species. Some specimens are from xerophytic scrub, while others are found in lowland forest. Microhabitat likely has a direct influence on the diversification of pholcids spiders, as was demonstrated by Eberle et al. (2018). Therefore, the morphological and molecular evidence suggest that *P.modestus* might be a species complex, as observed in *P.mysticus*. However, more male and female specimens from different populations are needed to confirm this assumption.

As Carstens et al. (2013) suggested, probing different methods or lines of evidence is necessary for properly implementing species delimitation. When the information and results are incongruent, it is better to be conservative about assumptions of species delimitation. In the case of some species in this study (e.g., *P.enaulus* and *P.xerophilus*), more detailed analyses of the morphological structures are necessary. Maybe including ultra-morphology, lineal morphology, or geometric morphometry in somatic features such as length legs, carapace shape, or even in sexual structures (Valdez-Mondragón et al. 2019). Lineal and geometric morphology has provided strong evidence for splitting species in cases where traditional morphology fails to delimit species. This has been demonstrated in araneomorph spiders (Planas and Ribera 2015; Valdez-Mondragón et al. 2019) and in brown recluse spiders of the genus *Loxosceles* (Solís-Catalán 2020). In this works, significant differences were found in carapace length, male palp shape, and length leg I among different species from the Canary Islands and central Mexico. *Physocyclusenaulus* is a widespread species from northern Mexico and southern United States. Valdez-Mondragón (2010) reported three different types of ventral apophyses in the female epigyne, which suggests that it might comprise a species complex rather than wide intraspecific morphological variation.

Although the number of described species in the genus *Physocyclus* has doubled in the last decade (Valdez-Mondragón 2010, 2013, 2014; Jiménez and Palacios-Cardiel 2013; Nolasco and Valdez-Mondragón 2020, 2022), the diversity of this genus in Mexico is still poorly known. Provinces such as the Sonoran and Chihuahuan Deserts have been poorly sampled, despite their arid and semiarid ecosystems being common habitats for the genus *Physocyclus*. Furthermore, cave habitats have been virtually unexplored in this genus, and will likely produce new troglophilic species (Valdez-Mondragón 2010, 2020; Huber et al. 2018).

In regard to the molecular methods used herein, each one presents its advantages and disadvantages. Barcoding methods (ABGD) can distinguish within-population differences caused by species divergences, analyzing the gaps of a data set and using it as a barcode to recognize different species (Puillandre et al. 2012; Rannala 2015; Rannala and Yang 2020). However, this method does not consider the rates of intra- and interspecific variation as an initial parameter, causing barcode values to vary among species groups and, in some cases, generating over splitting (Hickerson et al. 2006; Rannala 2015; Rannala and Yang 2020). The hierarchical integration of the ASAP method allows for many possible clusterings of terminals to be tested (Puillandre et al. 2021). However, it can offer different delimitation species hypothesis with similar asap-scores. In the case of *Physocyclus* spiders here, both methods offered a high congruence between themselves and the morphological evidence.

The coalescence method (GMYC) is robust because it uses an a priori ultrametric species tree, taking into account the groups formed in the topology. However, this method assumes that the lineages in each population coalesce before any speciation event occurs, implying the absence of incomplete lineage sorting and ignoring the coalescent process within populations of ancestral species (Rannala and Yang 2020). The bPTP method accommodates the use of large data sets with thousands of species and considers the rates of intra- and interspecific genetic variation. However, it does not take into account the stochastic fluctuations in the coalescent process among the different loci in large multi-locus analyses (Rannala and Yang 2020). The coalescent analyses for species delimitation (GMYC, bPTP) do not require reciprocal monophyly to delimit species (Knowles and Carstens 2007) and can incorporate statistical uncertainty in gene trees. They are based on Maximum Likelihood and Bayesian Inferences and not only on corrected genetic distances.

In conclusion, CO1 and 28S provide robust evidence for species-level delimitation in the genus *Physocyclus*, with high congruence among all methods. The genetic variability of ITS2 makes it an unreliable molecular marker for species delimitation on its own, however, it provides good information when used in combination with others mitochondrial and nuclear markers. Sexual morphological characters (male palps, male chelicerae, and female epigyne) are robust features for identifying and diagnosing *Physocyclus* species. However, in some cases, morphology alone is not enough to detect sister species, cryptic species, or even species complexes.

## References

[B1] Agnarsson I (2010) The utility of ITS2 in spider phylogenetics: Notes on prior work and an example from *Anelosimus*. The Journal of Arachnology 38(2): 377–382. 10.1636/B10-01.1

[B2] Álvarez-Padilla F Dimitrov D Giribet G Hormiga G (2009) Phylogenetic relationships of the spider family Tetragnathidae (Araneae, Araneoidea) based on morphological and DNA sequence data. Cladistics 25(2): 109–106. 10.1111/j.1096-0031.2008.00242.x 34879602

[B3] Arnedo MA Hormiga G Scharff N (2009) Higher-level phylogenetics of linyphiid spiders (Araneae, Linyphiidae) based on morphological and molecular evidence. Cladistics 25(3): 231–262. 10.1111/j.1096-0031.2009.00249.x 34879614

[B4] Astrin JJ Stueben PE (2008) Phylogeny in cryptic weevils: molecules, morphology and new genera of western Palaearctic *Cryptorhynchinae* (Coleoptera: Curculionidae). Invertebrate Systematics 22(5): 503–522. 10.1071/IS07057

[B5] Astrin JJ Huber BA Misof B Klütsch CFC (2006) Molecular taxonomy in pholcid spiders (Pholcidae: Araneae): evaluation of species identification methods using CO1 and 16S and rRNA. Zoologica Scripta 35(5): 441–457. 10.1111/j.1463-6409.2006.00239.x

[B6] Ballesteros JA Hormiga G (2018) Species delimitation of the North American orchard-spider *Leucaugevenusta* (Walckenaer, 1841) (Araneae, Tetragnathidae). Molecular Phylogenetics and Evolution 121: 183–197. 10.1016/j.ympev.2018.01.002 29337274

[B7] Barrett RDH Hebert PDN (2005) Identifying spiders through DNA barcodes. Canadian Journal of Zoology 83(3): 481–491. 10.1139/z05-024

[B8] Bickford D Lohman DJ Sodhi NS Ng PKL Meier R Winker L Ingram KK Das I (2007) Cryptic species as a window on diversity and conservation. Trends in Ecology & Evolution 22(3): 148–155. 10.1016/j.tree.2006.11.004 17129636

[B9] Bond JE (2004) Systematics of the Californian euctenizine spider genus *Apomastus* (Araneae: Mygalomorphae: Cyrtaucheniidae): the relationship between molecular and morphological taxonomy. Invertebrate Systematics 18(4): 361–376. 10.1071/IS04008

[B10] Bruvo-Mađarić B Huber BA Steinacher A Pass G (2005) Phylogeny of pholcid spiders (Araneae: Pholcidae): combined analysis using morphology and molecules. Molecular Phylogenetics and Evolution 37(3): 661–673. 10.1016/j.ympev.2005.08.016 16242969

[B11] Candia-Ramírez D Francke O (2020) Another stripe on the tiger makes no difference? Unexpected diversity in the widespread tiger tarantula *Davuspentaloris* (Araneae: Theraphosidae: Theraphosinae). Zoological Journal of the Linnean Society 192(1): 75–104. 10.1093/zoolinnean/zlaa107

[B12] Cao X Liu J Chen J Zheng G Kuntner M Agnarsson I (2016) Rapid dissemination of taxonomic discoveries based on DNA barcoding and morphology. Scientific Reports 6(1): e37066. 10.1038/srep37066 PMC5171852 27991489

[B13] Carstens BC Pelletier TA Reid NM Satler J (2013) How to fail at species delimitation. Molecular Ecology 22(17): 4369–4383. 10.1111/mec.12413 23855767

[B14] Correa-Ramírez MM Jiménez ML García-De León FJ (2010) Testing species boundaries in *Pardosasierra* (Araneae: Lycosidae). The Journal of Arachnology 38(3): 538–554. 10.1636/Sh09-15.1

[B15] Cruz-López JA Monjaraz-Ruedas R Francke OF (2019) Turning to the dark side: Evolutionary history and molecular species delimitation of a troglomorphic lineage of armoured harvestman (Opiliones: Stygnopsidae). Arthropod Systematics & Phylogeny 77(2): 285–302. 10.26049/ASP77-2-2019-0

[B16] DeSalle R Egan MG Siddall M (2005) The unholy trinity: Taxonomy, species delimitation and DNA barcoding. Philosophical Transactions of the Royal Society, London, Series B 360(1462): 1905–1916. 10.1098/rstb.2005.1722 PMC1609226 16214748

[B17] Dimitrov D Hormiga G (2011) An extraordinary new genus of spiders from Western Australia with an expanded hypothesis on the phylogeny of Tetragnathidae (Araneae). Zoological Journal of the Linnean Society 161(4): 735–768. 10.1111/j.1096-3642.2010.00662.x

[B18] Dimitrov D Astrin JJ Huber BA (2013) Pholcid spider molecular systematics revisited, with new insights into the biogeography and evolution of the group. Cladistics 29(2): 132–146. 10.1111/j.1096-0031.2012.00419.x 34814376

[B19] Drummond AJ Suchard MA Xie D Rambaut A (2012 (2002–2018) Bayesian phylogenetics with BEAUti and the BEAST 1.7. Molecular Biology and Evolution 29(8): 1969–1973. 10.1093/molbev/mss075 PMC3408070 22367748

[B20] Eberhard WG (1985) Sexual Selection and Animal Genitalia. Harvard University Press, Cambridge, Massachusetts, 244 pp. 10.4159/harvard.9780674330702

[B21] Eberhard WG Huber BA Rodríguez RL Briceno RD Salas I Rodríguez V (1998) One size fits all? Relationships between the size and degree of variation in genitalia and other body parts in twenty species of insects and spiders. Evolution 52(2): 415–431. 10.1111/j.1558-5646.1998.tb01642.x 28568329

[B22] Eberle J Dimitrov D Valdez-Mondragón A Huber BA (2018) Microhabitat change drives diversification in pholcid spiders. BMC Evolutionary Biology 18(1): 141. 10.1186/s12862-018-1244-8 30231864 PMC6145181

[B23] Folmer M Black W Lutz R Vrijenhoek R (1994) DNA primers for amplification of mitochondrial cytochrome c oxidase subunit I from diverse metazoan invertebrates. Molecular Marine Biology and Biotechnology 3: 294–299. 10.1603/00222585(2005)042[0756:MOBLIA]2.0.CO;2 7881515

[B24] Fox GE Pechman KR Woese CR (1977) Comparative cataloging of 16S ribosomal ribonucleic acid: Molecular approach to procaryotic systematics. International Journal of Systematic and Evolutionary Microbiology 27(1): 44–57. 10.1099/00207713-27-1-44

[B25] Fujisawa T Barraclough TG (2013) Delimiting species using single-locus data and the Generalized Mixed Yule Coalescent approach: A revised method and evaluation on simulated data sets. Systematic Biology 62(5): 707–724. 10.1093/sysbio/syt033 23681854 PMC3739884

[B26] Galtier N Nabholz B Glemin S Hurst GDD (2009) Mitochondrial DNA as a marker of molecular diversity: A reappraisal. Molecular Ecology 18(22): 4541–4550. 10.1111/j.1365-294X.2009.04380.x 19821901

[B27] Hall TA (1999) BioEdit: A user-friendly biological sequence alignment editor and analysis program for Windows 95/98/NT. Nucleic Acids Symposium Series 41: 95–98.

[B28] Hamilton CA Formanowicz DR Bond JE (2011) Species delimitation and phylogeography of *Aphonopelmahentzi* (Araneae, Mygalomorphae, Theraphosidae): Cryptic diversity in North American tarantulas. PLoS ONE 6(10): e26207. 10.1371/journal.pone.0026207 PMC3192178 22022570

[B29] Hamilton CA Hendrixson BE Brewer MS Bond JE (2014) An evaluation of sampling effects on multiple DNA barcoding methods leads to an integrative approach for delimiting species: A case study of the North American tarantula genus *Aphonopelma* (Araneae, Mygalomorphae, Theraphosidae). Molecular Phylogenetics and Evolution 71: 79–93. 10.1016/j.ympev.2013.11.007 24280211

[B30] Hazzi N Hormiga G (2021) Morphological and molecular evidence support the taxonomic separation of the medically important Neotropical spiders *Phoneutriadepilata* (Strand, 1909) and *P.boliviensis* (F.O. Pickard-Cambridge, 1897) (Araneae, Ctenidae). ZooKeys 1022(31): 13–50. 10.3897/zookeys.1022.60571 33762866 PMC7960689

[B31] Hebert PDN Cywinska A Ball SL deWaard JR (2003) Biological identifications through DNA barcodes. Proceedings of the Royal Society of London, Series B: Biological Sciences 270(1512): 313–321. 10.1098/rspb.2002.2218 PMC1691236 12614582

[B32] Hebert PDN Penton EH Burns JM Janzen DH Hallwachs W (2004) Ten species in one: DNA barcoding reveals cryptic species in the Neotropical skipper butterfly *Astraptesfulgerator*. Proceedings of the National Academy of Sciences of the United States of America 101(41): 14812–14817. 10.1073/pnas.0406166101 15465915 PMC522015

[B33] Hickerson MJ Meyer CP Moritz C (2006) DNA barcoding will often fail to discover new animal species over broad parameter space. Systematic Biology 55(5): 729–739. 10.1080/10635150600969898 17060195

[B34] Hillis DM Dixon MT (1991) Ribosomal DNA: molecular evolution and phylogenetic inference. Quarterly Review of Biology 66(4): 411–453. 10.1086/417338 1784710

[B35] Huber BA (2003) Rapid evolution and species-specificity of arthropod genitalia: Fact or artifact? Organisms, Diversity & Evolution 3(1): 63–71. 10.1078/1439-6092-00059

[B36] Huber BA (2011) Phylogeny and classification of Pholcidae (Araneae): An update. The Journal of Arachnology 39(2): 211–222. 10.1636/CA10-57.1

[B37] Huber BA Carvalho LS (2019) Filling the gaps: descriptions of unnamed species included in the latest molecular phylogeny of Pholcidae (Araneae). Zootaxa 4546(1): 001–096. 10.11646/zootaxa.4546.1.1 30790874

[B38] Huber BA Dimitrov D (2014) Slow genital and genetic but rapid non-genital and ecological differentiation in a pair of spider species (Araneae, Pholcidae). Zoologischer Anzeiger 253(5): 394–403. 10.1016/j.jcz.2014.04.001

[B39] Huber BA Pérez-González A (2001a) A new genus of pholcid spiders (Araneae: Pholcidae) endemic to Western Cuba, with a case of female genitalic dimorphism. American Museum Novitates 3329: 1–23. 10.1206/0003-0082(2001)329<0001:ANGOPS>2.0.CO;2

[B40] Huber BA Pérez-González A (2001b) Female genital dimorphism in a spider (Araneae: Pholcidae). The Zoological Society of London 255(3): 301–304. 10.1017/S095283690100139X

[B41] Huber BA Rheims CA Brescovit AD (2005) Speciation without changes in genital shape: a case study on Brazilian pholcid spiders (Araneae: Pholcidae). Zoologischer Anzeiger 243(4): 273–279. 10.1016/j.jcz.2004.12.001

[B42] Huber BA Eberle J Dimitrov D (2018) The phylogeny of pholcid spiders: A critical evaluation of relationships suggested by molecular data (Araneae, Pholcidae). ZooKeys 789: 51–101. 10.3897/zookeys.789.22781 PMC6193417 30344435

[B43] Ji Y Zhang D He L (2003) Evolutionary conservation and versatility of a new set of primers for amplifying the ribosomal internal transcribed spacer regions in insects and other invertebrates. Molecular Ecology Notes 3(4): 581–585. 10.1046/j.1471-8286.2003.00519.x

[B44] Jiménez ML Palacios-Cardiel CC (2013) A new species of *Physocyclus* (Araneae: Pholcidae) from Mexico. Zootaxa 3717(1): 96–99. 10.11646/zootaxa.3717.1.8 26176098

[B45] Jörger KM Schrödl M (2013) How to describe a cryptic species? practical challenges of molecular taxonomy. Frontiers in Zoology 10(1): 59. 10.1186/1742-9994-10-59 24073641 PMC4015967

[B46] Kapli P Lutteropp S Zhang J Kobert K Pavlidis P Stamatakis A Flouri T (2017) Multi-rate Poisson tree processes for single-locus species delimitation under maximum likelihood and Markov chain Monte Carlo. Bioinformatics 33: 1630–1638. 10.1093/bioinformatics/btx025 28108445 PMC5447239

[B47] Katoh K Toh H (2008) Recent developments in the MAFFT multiple sequence alignment program. MAFFT version 7. Briefings in Bioinformatics 4(4): 286–298. 10.1093/bib/bbn013 [accessed 13 April 2022] 18372315

[B48] Knowles LL Carstens BC (2007) Delimiting species without monophyletic gene trees. Systematic Biology 56(6): 887–895. 10.1080/10635150701701091 18027282

[B49] Kumar S Stecher G Tamura K (2016) MEGA7: Molecular evolutionary genetics analysis v.7.0 for bigger datasets. Molecular Biology and Evolution 33(7): 1870–1874. 10.1093/molbev/msw054 27004904 PMC8210823

[B50] Letunic I Bork P (2021) Interactive Tree Of Life (iTOL) v5: An online tool for phylogenetic tree display and annotation. Nucleic Acids Research 49(1): 293–296. 10.1093/nar/gkab301 PMC8265157 33885785

[B51] Luo A Ling C Ho YM Zhu CD (2018) Comparison of Methods for Molecular Species Delimitation Across a Range of Speciation Scenarios. Systematic Biology 67(5): 830–846. 10.1093/sysbio/syy011 29462495 PMC6101526

[B52] Monaghan MT Wild R Elliot M Fujisawa T Balke M Inward DJ Vogler AP (2009) Accelerated species inventory on Madagascar using coalescent-based models of species delineation. Systematic Biology 58(3): 298–311. 10.1093/sysbio/syp027 20525585

[B53] Montes de Oca L D’Elía G Pérez-Miles F (2015) An integrative approach for species delimitation in the spider genus *Grammostola* (Theraphosidae, Mygalomorphae). Zoologica Scripta 45(3): 322–333. 10.1111/zsc.12152

[B54] Navarro-Rodríguez I Valdez-Mondragón A (2020) Description of a new species of *Loxosceles* Heineken & Lowe (Araneae, Sicariidae) recluse spiders from Hidalgo, Mexico, under integrative taxonomy: Morphological and DNA barcoding data (CO1+ITS2). European Journal of Taxonomy 704(704): 1–30. 10.5852/ejt.2020.704

[B55] Newton LG Starrett J Hendrixson BE Derkarabetian S Bond JE (2020) Integrative species delimitation reveals cryptic diversity in the southern Appalachian *Antrodiaetusunicolor* (Araneae: Antrodiaetidae) species complex. Molecular Ecology 29(12): 2269–2287. 10.1111/mec.15483 32452095

[B56] Nolasco S Valdez-Mondragón A (2020) On the daddy long-legs spiders of the genus *Physocyclus* (Araneae: Pholcidae) from Mexico: description of a new species from the Baja California Peninsula. Revista Mexicana de Biodiversidad 91(1): 913316. 10.22201/ib.20078706e.2020.91.3316

[B57] Nolasco S Valdez-Mondragón A (2022) Four new species of the spider genus *Physocyclus* Simon, 1893 (Araneae: Pholcidae) from Mexico, with updated taxonomic identification keys. European Journal of Taxonomy 813: 173–206. 10.5852/ejt.2022.813.1739

[B58] Ortiz D Francke O (2016) Two DNA barcodes and morphology for multi-method species delimitation in *Bonnetina* tarantulas (Araneae: Theraphosidae). Molecular Phylogenetics and Evolution 101: 176–193. 10.1016/j.ympev.2016.05.003 27150350

[B59] Planas E Ribera C (2014) Uncovering overlooked island diversity: colonization and diversification of the medically important spider genus *Loxosceles* (Arachnida: Sicariidae) on the Canary Islands. Journal of Biogeography 41(7): 1255–1266. 10.1111/jbi.12321

[B60] Planas E Ribera C (2015) Description of six new species of *Loxosceles* (Araneae: Sicariidae) endemic to the Canary Islands and the utility of DNA barcoding for their fast and accurate identification. Zoological Journal of the Linnean Society 174(1): 47–73. 10.1111/zoj.12226

[B61] Pons J Barraclough TG Gomez-Zurita J Cardoso A Duran DP Hazell S Kamoun S Sumlin WD Vogler AP (2006) Sequence based species delimitation for the DNA taxonomy of undescribed insects. Systematic Biology 55(4): 595–609. 10.1080/10635150600852011 16967577

[B62] Puillandre N Lambert A Brouillet S Achaz G (2012) ABGD, Automatic Barcode Gap Discovery for primary species delimitation. Molecular Ecology 21(8): 1864–1877. 10.1111/j.1365-294X.2011.05239.x 21883587

[B63] Puillandre N Brouillet S Achaz G (2021) ASAP: Assemble species by automatic partitioning. Molecular Ecology Resources 21(2): 609–620. 10.1111/1755-0998.13281 33058550

[B64] Rambaut A Drummond AJ (2003–2013) . TRACER, MCMC trace analysis tool. Version 1.6. Institute of Evolutionary Biology, University of Edinburgh, Edinburgh, Department of Computer Science, University of Auckland, Auckland.

[B65] Rannala B (2015) The art and science of species delimitation. Current Zoology 61(5): 846–853. 10.1093/czoolo/61.5.846

[B66] Rannala B Yang Z (2020) Species Delimitation. Phylogenetics in the Genomic Era, No commercial publisher, Authors open access book, 5.5:1–5.5:18. https://hal.archives-ouvertes.fr/hal-02536468

[B67] Rozen S Skaletsky JH (2000) Primer3 on the www for general users and for biologist programmers. In: Krawertz S, Misener S (Eds) Bioinformatics Methods and Protocols: Methods in Molecular Biology. Humana Press, Totowa, N, J. 365–386. 10.1385/1-59259-192-2:365 10547847

[B68] Solís-Catalán KP (2020) Análisis morfométrico de estructuras sexuales y somáticas de las especies mexicanas de arañas del género *Loxosceles* Heineken y Lowe (Araneae, Sicariidae) del Centro-Occidente de México. Tesis de Maestría en Ciencias. Universidad Autónoma de Tlaxcala.

[B69] Tautz D Arctander P Minelli A Thomas RH Vogler AP (2003) A plea for DNA taxonomy. Trends in Ecology & Evolution 18(2): 70–74. 10.1016/S0169-5347(02)00041-1

[B70] Tyagi K Kumar V Kundu S Pakrashi A Prasad P Caleb JTD Chandra K (2019) Identification of Indian spiders through DNA barcoding: Cryptic species and species complex. Scientific Reports 9(1): 14033. 10.1038/s41598-019-50510-8 31575965 PMC6773733

[B71] Valdez-Mondragón A (2010) Revisión taxonómica de *Physocyclus* Simon, 1983 (Araneae: Pholcidae) con la descripción de especies nuevas de México. Revista Iberica de Aracnologia 18: 3–80.

[B72] Valdez-Mondragón A (2013) Morphological phylogenetic analysis of the spider genus *Physocyclus* (Araneae: Pholcidae). The Journal of Arachnology 41(2): 184–196. 10.1636/K12-33.1

[B73] Valdez-Mondragón A (2014) A reanalysis of the morphological phylogeny of the spider genus *Physocyclus* Simon, 1983 (Araneae: Pholcidae) with the description of a new species and description of the female of *Physocyclusparedesi* Valdez-Mondragón from México. Zootaxa 3866: 202–220. 10.11646/zootaxa.3866.2.2 25283655

[B74] Valdez-Mondragón A (2020) COI mtDNA barcoding and morphology for species delimitation in the spider genus *Ixchela* Huber (Araneae: Pholcidae), with the description of two new species from Mexico. Zootaxa 4747(1): 054–076. 10.11646/zootaxa.4747.1.2 32230118

[B75] Valdez-Mondragón A Cortez-Roldán M (2021) COI mtDNA barcoding and morphology for the description of a new species of ricinuleid of the genus *Pseudocellus* (Arachnida: Ricinulei: Ricinoididae) from El Triunfo Biosphere Reserve, Chiapas, Mexico. European Journal of Taxonomy 778: 1–25. 10.5852/ejt.2021.778.1563

[B76] Valdez-Mondragón A Francke OF (2015) Phylogeny of the spider genus *Ixchela* Huber, 2000 (Araneae: Pholcidae) based on morphological and molecular evidence (CO1 and 16S), with a hypothesized diversification in the Pleistocene. Zoological Journal of the Linnean Society 175(1): 20–58. 10.1111/zoj.12265

[B77] Valdez-Mondragón A Navarro-Rodríguez CI Solís-Catalán KP Cortez-Roldán MR Juárez-Sánchez AR (2019) Under an integrative taxonomic approach: The description of a new species of the genus *Loxosceles* (Araneae, Sicariidae) from Mexico City. ZooKeys 892: 93–133. 10.3897/zookeys.892.39558 31824205 PMC6892964

[B78] Wayne L Brenner D Colwell R Grimont P Kandler O Krichevsky M Moore L Moore W Murray R Stackebrandt E Starr MP Truper HG (1987) Report of the ad hoc committee on reconciliation of approaches to bacterial systematics. International Journal of Systematic and Evolutionary Microbiology 37(4): 463–464. 10.1099/00207713-37-4-463

[B79] Wilson KH (1995) Molecular biology as a tool for taxonomy. Clinical Infectious Diseases 20(Supplement_2): 117–121. 10.1093/clinids/20.Supplement_2.S117 7548531

[B80] WSC [World Spider Catalog] (2022) World Spider Catalog. Version 22.0. Natural History Museum Bern. 10.24436/2 [accessed on august 16, 2022]

[B81] Zhang Y Li S (2014) A spider species complex revealed high cryptic diversity in South Chine caves. Molecular Phylogenetics and Evolution 79: 353–358. 10.1016/j.ympev.2014.05.017 24994029

[B82] Zhang J Kapli P Pavlidis P Stamatakis A (2013) A general species delimitation method with applications to phylogenetic placements. Bioinformatics 29(22): 2869–2876. 10.1093/bioinformatics/btt499 23990417 PMC3810850

